# Cell type and sex specific mitochondrial phenotypes in iPSC derived models of Alzheimer’s disease

**DOI:** 10.3389/fnmol.2023.1201015

**Published:** 2023-08-08

**Authors:** Kaitlin Flannagan, Julia A. Stopperan, Brittany M. Hauger, Benjamin R. Troutwine, Colton R. Lysaker, Taylor A. Strope, Vivien Csikos Drummond, Caleb A. Gilmore, Natalie A. Swerdlow, Julia M. Draper, Cynthia M. Gouvion, Jay L. Vivian, Mohammad Haeri, Russell H. Swerdlow, Heather M. Wilkins

**Affiliations:** ^1^University of Kansas Alzheimer's Disease Research Center, University of Kansas Medical Center, Kansas City, KS, United States; ^2^Department of Biochemistry and Molecular Biology, University of Kansas Medical Center, Kansas City, KS, United States; ^3^Transgenic and Gene Targeting Facility, University of Kansas Medical Center, Kansas City, KS, United States; ^4^Department of Pediatrics, University of Kansas Missouri-Kansas City School of Medicine, Kansas City, KS, United States; ^5^Institute for Reproduction and Perinatal Research, University of Kansas Medical Center, Kansas City, KS, United States; ^6^Department of Pathology & Laboratory Medicine, University of Kansas Medical Center, Kansas City, KS, United States; ^7^Department of Molecular and Integrative Physiology, University of Kansas Medical Center, Kansas City, KS, United States; ^8^Department of Neurology, University of Kansas Medical Center, Kansas City, KS, United States

**Keywords:** mitochondria, Alzheimer’s disease, neuron, astrocyte, induced pluripotent stem cell

## Abstract

**Introduction:**

Mitochondrial dysfunction is observed in Alzheimer’s disease (AD). Altered mitochondrial respiration, cytochrome oxidase (COX) Vmax, and mitophagy are observed in human subjects and animal models of AD. Models derived from induced pluripotent stem cells (iPSCs) may not recapitulate these phenotypes after reprogramming from differentiated adult cells.

**Methods:**

We examined mitochondrial function across iPSC derived models including cerebral organoids, forebrain neurons, and astrocytes. iPSCs were reprogrammed from fibroblasts either from the University of Kansas Alzheimer’s Disease Research Center (KU ADRC) cohort or purchased from WiCell. A total of four non-demented and four sporadic AD iPSC lines were examined. Models were subjected to mitochondrial respiration analysis using Seahorse XF technology, spectrophotometric cytochrome oxidase (COX) Vmax assays, fluorescent assays to determine mitochondrial mass, mitochondrial membrane potential, calcium, mitochondrial dynamics, and mitophagy levels. AD pathological hallmarks were also measured.

**Results:**

iPSC derived neurons and cerebral organoids showed reduced COX Vmax in AD subjects with more profound defects in the female cohort. These results were not observed in astrocytes. iPSC derived neurons and astrocytes from AD subjects had reduced mitochondrial respiration parameters with increased glycolytic flux. iPSC derived neurons and astrocytes from AD subjects showed sex dependent effects on mitochondrial membrane potential, mitochondrial superoxide production, and mitochondrial calcium. iPSC derived neurons from AD subjects had reduced mitochondrial localization in lysosomes with sex dependent effects on mitochondrial mass, while iPSC derived astrocytes from female AD subjects had increased mitochondrial localization to lysosomes. Both iPSC derived neurons and astrocytes from AD subjects showed altered mitochondrial dynamics. iPSC derived neurons had increased secreted Aβ, and sex dependent effects on total APP protein expression. iPSC derived astrocytes showed sex dependent changes in GFAP expression in AD derived cells.

**Conclusion:**

Overall, iPSC derived models from AD subjects show mitochondrial phenotypes and AD pathological hallmarks in a cell type and sex dependent manner. These results highlight the importance of sex as a biological variable in cell culture studies.

## Introduction

Mitochondrial dysfunction is observed in AD across numerous tissues. Reductions in cytochrome oxidase (COX) Vmax have been observed in brain, blood, and skin from AD subjects (Parker and Parks, 1995; Bosetti et al., 2002; Cardoso et al., 2004; Swerdlow et al., 2017). Across models of AD, including mouse and cell models, alterations to mitochondrial oxygen consumption, reactive oxygen species (ROS), and mitophagy are observed (Arduino et al., 2015; Guo et al., 2017; Swerdlow et al., 2017). Mitochondrial phenotypes in specific cell types are difficult to study in postmortem human and animal models of AD. However, induced pluripotent stem cells (iPSC) have emerged as a tool to study AD and understand cell type specific changes in mitochondrial function.

iPSC derived neurons have mitochondrial dysfunction independent of AD pathological hallmarks. Prior studies indicate iPSC derived neurons from sporadic AD patients show increased oxidative stress and mtDNA damage/mutations. These observations were independent of AD pathological hallmarks including tau neurofibrillary tangles and Aβ plaques (Birnbaum et al., 2018). iPSC derived neurons from sporadic and familial AD subjects show impaired mitophagy linked to lysosomal defects (Martin-Maestro et al., 2017). Further studies have examined overexpression of APP and tau in iPSC derived neurons, which impaired mitophagy. Due to changes observed in iPSC derived neurons, they have been used to test potential therapeutics to remedy mitophagy failure in AD (Martin-Maestro et al., 2019a,b).

iPSC derived neurons often show elevated Aβ in addition to increased mtDNA mutation burden and altered mitochondrial function (Lee et al., 2022). Familial AD derived neurons also recapitulate increased Aβ and tau pathology/accumulation with reduced mitochondrial trafficking (Li et al., 2019). Studies comparing iPSC derived neurons with postmortem brain tissue from sporadic AD subjects have identified overlapping proteomic profiles between sample types. Mitophagy and autophagy were major implicated pathways between iPSC derived neurons and postmortem brain samples (Verheijen et al., 2022). Overall iPSC derived models likely recapitulate disease mechanisms observed in postmortem human tissue and could be a relevant model for testing therapeutics.

Studies examining iPSC derived astrocytes in the context of mitochondrial function are limited. iPSC derived astrocytes treated with Aβ resulted in mitochondrial dysfunction and a shift in bioenergetic pathways to glycolysis and fatty acid oxidation (Zysk et al., 2023). A separate study also reported changes in mitochondrial energy metabolism and mitochondrial transcriptome in iPSC derived astrocytes (Ryu et al., 2021). Beyond iPSC derived astrocytes, cerebral organoids are potential models to recapitulate cell-to-cell interactions, however limited data are available examining mitochondrial dysfunction in these models.

Here, we compared iPSC neurons and astrocytes from the same individuals to examine cell type specific changes in mitochondrial function from non-demented (ND) and sporadic AD (AD) donors. We leveraged age and sex matched iPSC models from the University of Kansas Alzheimer’s Disease Research Center (KU ADRC) clinical cohort and WiCell.

## Methods

### iPSC source and reprogramming

iPSCs were reprogrammed from dura fibroblasts obtained at the University of Kansas Alzheimer’s Disease Research Center (KU ADRC) or purchased from WiCell. KU ADRC fibroblast donors were members of the Clinical Cohort, who consented to donation upon death and approval from an ethical standards committee to conduct this study was received. The studies involving human participants were reviewed and approved by the University of Kansas Medical Center Institutional Review Board. Banked tissue is de-identified by the KUADRC Neuropathology Core to eliminate identifying information. Reprogramming was completed using the Sendai Virus, CytoTune-iPS 2.0 *Sendai Reprogramming* Kit from ThermoFisher. iPSC were age, sex, and diagnosis matched (Table 1). For iPSCs derived from the KU ADRC cohort, ND or AD were diagnosed at autopsy neuropathological examination as outlined in the NACC Neuropathology Coding Guidebook (N.S. Committee, 2020).

**Table 1:** iPSC demographics.

iPSC identifier	Sex	Diagnosis
ADC37	Female	AD
UCSD231i-SAD1-3	Female	AD
ADC9	Male	AD
UCSD234i-SAD2-3	Male	AD
ADC8	Female	ND
UCSD067i-19-1	Female	ND
UCSD223i-NDC1-1	Male	ND
ADC25	Male	ND

iPSC demographics including age, sex, and diagnosis status. ND, non-demented; AD, Alzheimer’s disease. Average age for female ND = 80.5 years, female AD = 76 years, male ND = 87, and male AD = 79.5.

### iPSC neural progenitor cell differentiation

iPSCs were differentiated into neural progenitor cells (NPCs) using STEMDiff Neural Induction Medium (NIM). iPSCs were placed into a single cell suspension in NIM with SMADi/ROCKi (SMAD inhibitor and ROCK Inhibitor) in an AggreWell 800 plate. Embryoid bodies were cultured in the AggreWell plate for 5 days with NIM/SMADi partial medium changes daily. Embryoid bodies were then plated onto Matrigel coated plates and fed daily with NIM/SMADi medium until day 12 to allow neural rosette formation. Neural rosettes were selected using Neural Rosette Selection Reagent (StemCell Tech) and plated onto Matrigel coated dishes with NIM/SMADi. Media was changed daily for 7 days, after which neural progenitor cells (NPC) were cryopreserved and split into defined Neural Progenitor Medium (StemCell Tech).

### iPSC forebrain neuronal differentiation

NPCs were plated onto PLO/Laminin coated dishes in neural progenitor medium. The following day media was changed to StemDiff Forebrain Neural Differentiation Medium (StemCell Tech). Media was changed daily for 7 days (Muratore et al., 2014a,b). Following which cells were plated onto PLO/laminin coated dishes in defined Brain Phys Medium (with N2A, SM1, BDNF, GDNF, cAMP, and ascorbic acid) for neuronal maturation. Neurons were matured for 7–10 days and used for downstream experiments.

### iPSC astrocyte differentiation

NPCs were plated onto Matrigel coated dishes in neural progenitor medium. The following day cells were placed in astrocyte differentiation medium consisting of DMEM (high glucose, with glutamine, no pyruvate), B27, 1% FBS, glutamine, bFGF, CNTF, BMP8, Activin A, heregulin 1b, and IGF1. Medium was changed every other day and cells were passaged as needed (Shaltouki et al., 2013). After 30 days or approximately 5–6 passages astrocytes were used in downstream experiments.

### Cerebral organoid generation

Cerebral organoids were made using iPSC’s and StemDiff Cerebral Organoid kits. The iPSC’s were briefly placed into single cell suspensions with ROCKi in embryoid body formation plates. Embryoid bodies expanded for 7 days and were then embedded into Matrigel droplets. The organoids matured for 90 days.

### Vmax enzyme assays

We used the Complex IV Human Enzyme Activity Microplate Assay Kit from Abcam (ab109909). We followed the manufacturer’s protocol. All Vmax rates were normalized to protein content using a BCA protein assay (ThermoFisher).

### Seahorse analysis

To interrogate electron transport chain (ETC) function cells were plated in a seahorse XF96 microplate at 30,000 cells per well and allowed to adhere/mature. Cells were then placed in 180 uL of MAS buffer with 0.1 nM PMP reagent and 10 mM pyruvate, 5 mM malate, 4 mM ADP. Cartridge injections of drugs were as follows; A. 5 mM succinate and 2 μM rotenone, B. 4 μM Antimycin A. C. 0.5 mM TMPD and 1 mM ascorbic acid, D. 50 mM Sodium Azide. Oxygen consumption rates (OCR) were measured following a 1 min mix after addition of injections and measured over 2 min two times.

To interrogate glycolysis flux 30,000 cells per well were plated in an XF cell culture microplate and allowed to adhere/mature. Medium was changed to glycolysis stress test medium (serum-free, pyruvate-free, glucose-free, buffer-free DMEM). The plate was then placed in the Cytation 1 Cell Imaging MultiMode Reader from BioTek for brightfield images (37°C) for 45 min. Cartridge injections of drugs were as follows; A. 10 mM glucose, B. 1 μM Oligomycin with 10 μg/mL Hoechst, C. 100 mM 2-deoxy-glucose, and D. 1 μM rotenone and antimycin A. OCR and extracellular acidification rates (ECAR) were measured following a 1 min mix after addition of injections and measured over 2 min three times. Following the seahorse run the plate was returned to the Cytation 1 Cell Imaging MultiMode Reader from BioTek for automated cell counting. Each well was normalized to cell count.

To interrogate mitochondrial flux 30,000 cells per well were plated in an XF cell culture microplate and allowed to adhere/mature. Medium was changed to mitochondrial stress test medium (serum-free, pyruvate-free,buffer-free DMEM). The plate was then placed in the Cytation 1 Cell Imaging MultiMode Reader from BioTek for brightfield images (37°C) for 45 min. Cartridge injections of drugs were as follows; A. 1 μM Oligomycin with 10 μg/mL Hoechst, B. 0.25 μM FCCP, C. 0.25 μM FCCP, D. 1 μM rotenone and antimycin A. OCR and extracellular acidification rates (ECAR) were measured following a 1 min mix after addition of injections and measured over 2 min three times. Following the seahorse run the plate was returned to the Cytation 1 Cell Imaging MultiMode Reader from BioTek for automated cell counting. Each well was normalized to cell count.

### Mitochondrial dyes

These assays were completed independently in Corning 96-well plates. Mitochondrial membrane potential was determined using tetramethylrhodamine, ethyl ester (TMRE from ThermoFisher) at a concentration of 200 nM. Mitochondrial superoxide was measured using MitoSox Red (ThermoFisher) at a concentration of 500 nM. Hydrogen peroxide was measured using amplex ultra red at a concentration of 50 μM with 0.1 U/mL horseradish peroxidase (HRP). Hoechst was added to a final concentration of 10 μg/mL. Cells were incubated with dyes for 30 min and washed two times with Hank’s Balanced Buffer Solution (HBSS with Ca2+ and Mg2+). Fluorescence was read using a Tecan plate reader. Each dye intensity was normalized to Hoechst intensity. Representative images were collected using a Cytation 1 Cell Imaging MultiMode Reader from BioTek.

### Mitochondrial and total calcium

These assays were completed independently in Corning 96-well plates. Cells were loaded with 1 μM Rhod2AM or Fura2AM with 10 μg/mL for 30 min. Cells were washed two times with HBSS. Fluorescence was read using a Tecan plate reader. Each dye intensity was normalized to Hoechst intensity.

### Mitochondrial turnover and mitophagy

These assays were completed independently in Corning 96-well plates. Adenoviral constructs encoding MitoTimer and EGFP-COX8 were used in independent experiments. Cells were infected with adenoviral particles at an MOI of 10 for 48 h. Following which cells were stained with Hoechst was added to a final concentration of 10 μg/mL and washed with HBSS two times. Cells were imaged using a Cytation 1 Cell Imaging MultiMode Reader from BioTek. All data were normalized to cell number in each image. Red and green spots were counted per cell for MitoTimer, and red only spots were counted for EGFP-COX8 as previously described (Ma and Ding, 2021).

### Mitochondrial dynamics

These assays were completed independently in Corning 96-well plates. MitoTracker Red was used at a concentration of 50 nM. Hoechst was added to a final concentration of 10 μg/mL. Cells were incubated with dyes for 30 min and washed two times with Hank’s Balanced Buffer Solution (HBSS with Ca2+ and Mg2+). Fluorescence was read using a Tecan plate reader and images were collected using a Cytation 1 Cell Imaging MultiMode Reader from BioTek. Dye intensity was normalized to Hoechst intensity. For mitochondrial dynamic analysis, including branch number, junction number, and maximum branch length; we followed the protocol outlined in Valente et al. (2017).

### Aβ ELISA

Media protein was concentrated using ice-cold 100% acetone at a 2:1 ratio. Media samples were incubated in acetone for a minimum of 30 min at −20°C. Samples were sedimented by centrifugation at 5,000 × g for 5 min followed by a 70% ethanol wash. Samples were re-suspended in 8 M urea. Media samples were used in downstream ELISA assays. Aβ40 and Aβ42 were measured using human ELISA assays from ThermoFisher with samples diluted 1:5. All data were normalized to the protein content of the samples using a BCA protein assay (ThermoFisher).

### Western blots

Whole cell lysates were generated using RIPA buffer with protease and phosphatase inhibitors (Sigma and ThermoFisher). Briefly, an equal amount of protein was resolved via SDS-PAGE on Criterion TGX gels 4–15% (BioRad). Gels were transferred to PVDF membranes and blocked with 5% BSA in PBST. Primary antibodies were incubated overnight at 4°C followed by three washes with PBST. Secondary antibodies (ThermoFisher) were incubated at room temperature for 1 hour. Gels were imaged using WestFemto ECL (ThermoFisher) and a BioRad ChemiDoc XRS imaging system. Loading control was total protein stained using AmidoBlack (Sigma). Primary antibodies include amyloid precursor protein (APP), cytochrome oxidase 4 isoform 1 (COX41I), dynamin related protein 1 (DRP1), pDRP1 (Ser637), glial fibrillary acidic protein (GFAP), mitofusin 1 (MFN1) (all antibodies purchased from Abcam or ThermoFisher).

### qPCR

RNA was isolated using Trizol and phenol/chlorofom extraction. cDNA synthesis was performed using 1 μg of RNA and an iScript cDNA synthesis kit (BioRad). qPCR was performed using Taqman assays and reagents against ActB, GFAP, and MAP2 on a QuantStudio 5 platform (ThermoFisher).

### Data analysis

All experiments were completed at least three different sets of differentiated cells. Data were summarized by means and standard errors. To compare means between three or more groups, we used one-way ANOVA followed by Fisher’s least significant difference (LSD) *post hoc* testing. To compare means between two groups we used two-way, unpaired Student’s *t*-tests. Statistical tests were performed using Prism/Graph pad. *p*-values less than 0.05 were considered statistically significant.

## Results

**Figure 1:**
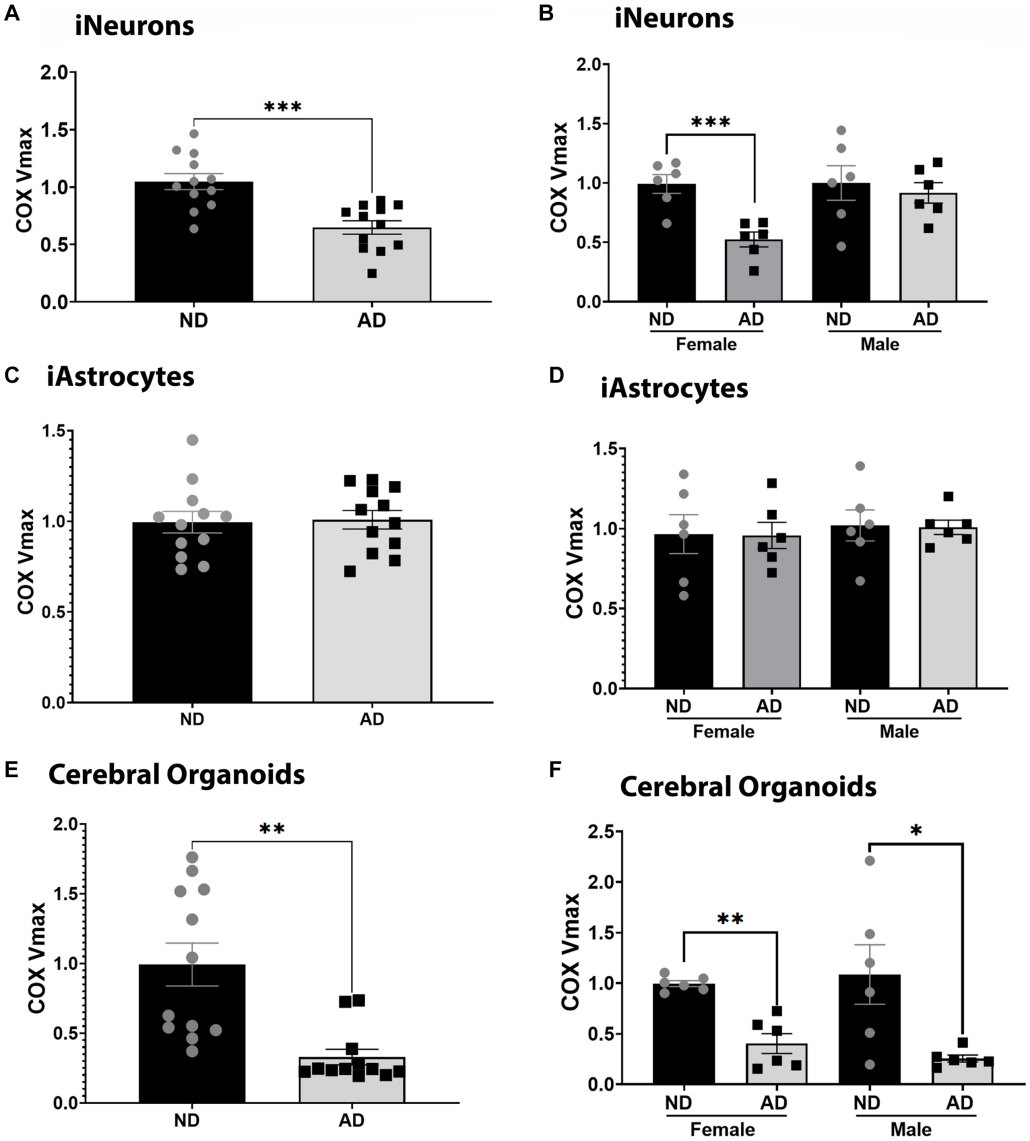
COX Vmax in iPSC models. **(A)** COX Vmax measured in ND and AD iPSC derived neurons; data are normalized to ND. **(B)** COX Vmax among female and male groups of ND and AD iPSC derived neurons, data are normalized to ND. **(C)** COX Vmax measured in ND and AD iPSC derived astrocytes; data are normalized to ND. **(D)** COX Vmax among female and male groups of ND and AD iPSC derived astrocytes, data are normalized to ND. **(E)** COX Vmax measured in ND and AD iPSC derived cerebral organoids; data are normalized to ND. **(F)** COX Vmax among female and male groups of ND and AD iPSC derived cerebral organoids, data are normalized to ND. ND, non-demented; AD, Sporadic Alzheimer’s disease. * Indicates *p* ≤ 0.05, *** indicates *p* ≤ 0.001, **** indicates *p* ≤ 0.0001, and ns indicates non-significant.

We examined COX Vmax across iPSC models. iPSC derived neurons (iNeurons) derived from AD subjects had reduced COX Vmax (Figure 1A) and this was driven by cells derived from female subjects (Figure 1B). iPSC derived astrocytes (iAstrocytes) showed no change in COX Vmax (Figures 1C,D). However, iPSC derived cerebral organoids showed reduced COX Vmax (Figure 1E) regardless of sex (Figure 1F). To determine if these observations were due to a change in expression of COX components, we examined the protein expression of COX4I1. There was no change in COX4I1 protein expression for any model type (data not shown).

**Figure 2:**
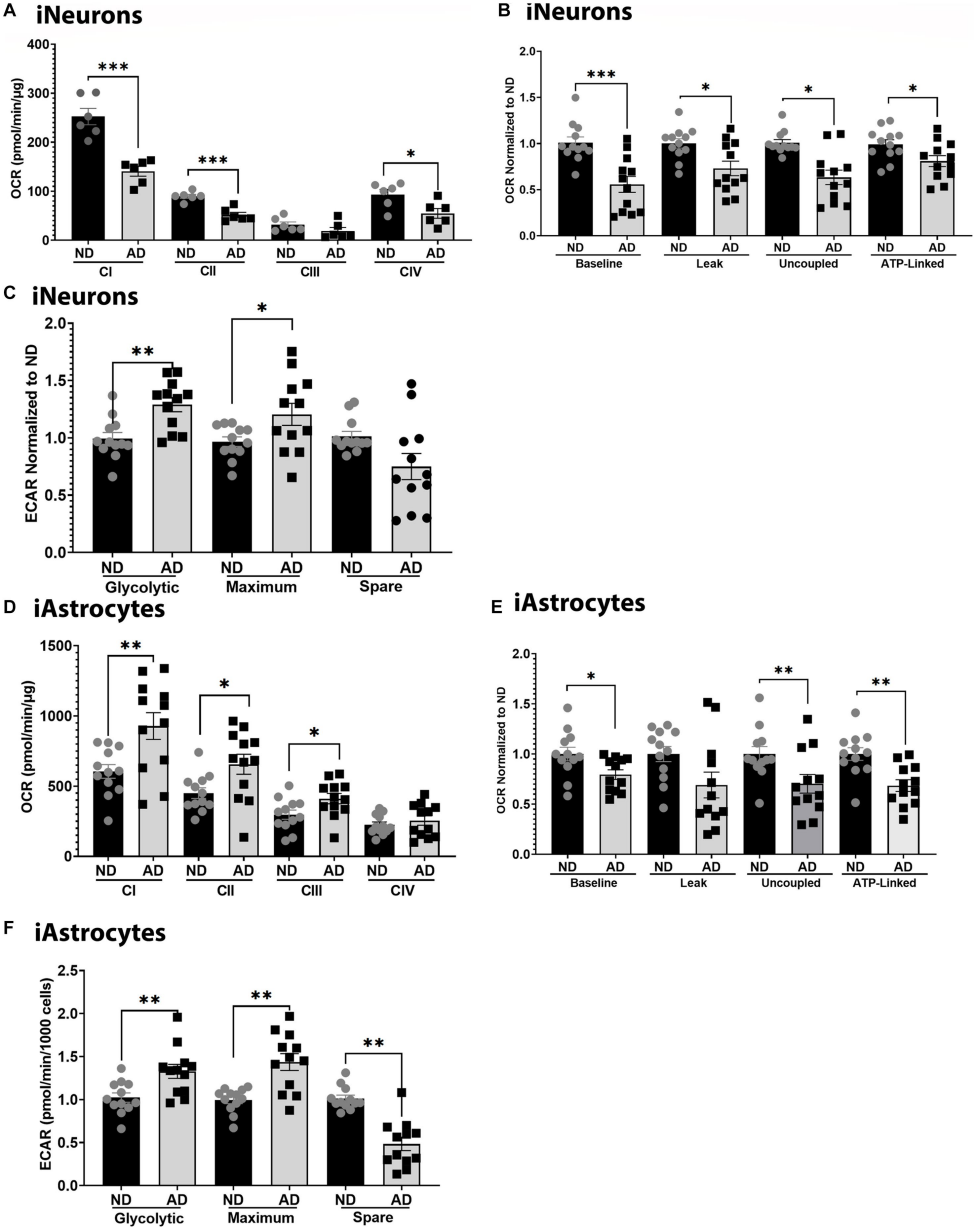
Mitochondrial respiration in iPSC models. **(A)** ETC flux analysis in ND and AD iPSC derived neurons; data are normalized to ND. **(B)** Mitochondrial stress test analysis in ND and AD iPSC derived neurons, data are normalized to ND. **(C)** Glycolytic flux analysis in ND and AD iPSC derived neurons; data are normalized to ND. **(D)** ETC flux analysis in ND and AD iPSC derived astrocytes; data are normalized to ND. **(E)** Mitochondrial stress test analysis in ND and AD iPSC derived astrocytes, data are normalized to ND. **(F)** Glycolytic flux analysis in ND and AD iPSC derived astrocytes; data are normalized to ND. ND, non-demented; AD, Sporadic Alzheimer’s disease. * Indicates *p* ≤ 0.05, ** indicates *p* ≤ 0.01, *** indicates *p* ≤ 0.001, **** indicates *p* ≤ 0.0001, and ns indicates non-significant.

To further examine mitochondrial function, we used seahorse assays to examine mitochondrial respiration, glycolytic flux, and ETC function. Seahorse analysis of ETC function showed that iNeurons had reduced flux at complexes I, II, and IV (Figure 2A). iNeurons also showed reduced baseline, uncoupled, and ATP-linked mitochondrial respiration (Figure 2B) with increased glycolytic flux (Figure 2C). Comparatively, iAstrocytes had increased ETC flux at complexes I-III, with reduced mitochondrial respiration at baseline, uncoupled, and ATP-linked respiration (Figures 2D,E). Glycolytic flux was also increased in iAstrocytes (Figure 2F). Seahorse tracings are shown in Supplementary Figure S2.

**Figure 3:**
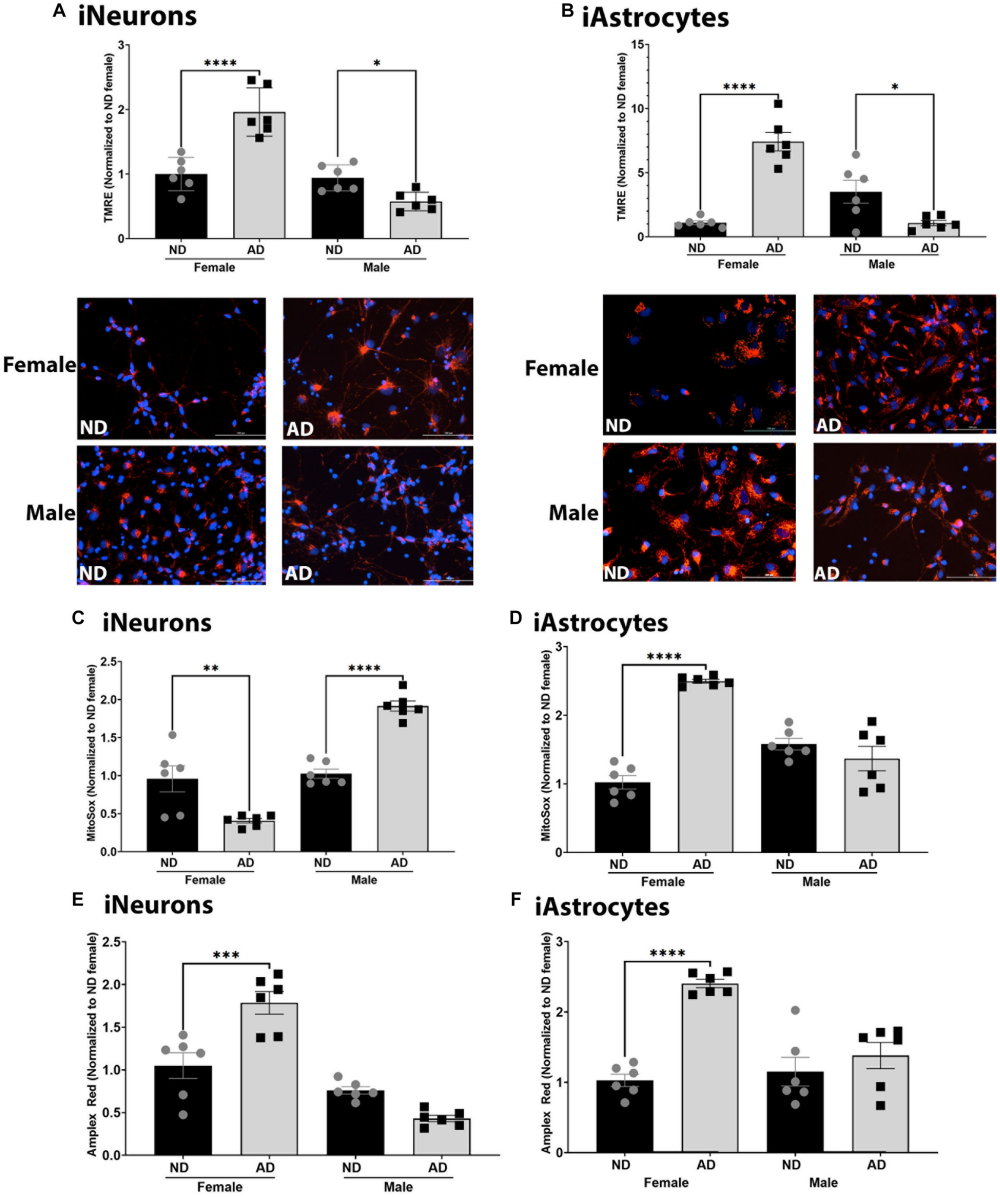
Mitochondrial membrane potential and ROS in iPSC derived models. **(A)** TMRE/Hoechst among female and male groups of ND and AD iPSC derived neurons, data are normalized to female ND. Representative images are shown. **(B)** TMRE/Hoechst among female and male groups of ND and AD iPSC derived astrocytes, data are normalized to female ND. Representative images are shown. **(C)** MitoSox/Hoechst among female and male groups of ND and AD iPSC derived neurons, data are normalized to female ND. **(D)** MitoSox/Hoechst among female and male groups of ND and AD iPSC derived astrocytes, data are normalized to female ND. **(E)** Amplex Red/Hoescht among female and male groups of ND and AD iPSC derived neurons, data are normalized to female ND. **(F)** Amplex Red/Hoescht among female and male groups of ND and AD iPSC derived astrocytes, data are normalized to female ND. ND, non-demented; AD, Alzheimer’s disease. * Indicates *p* ≤ 0.05, ** indicates *p* ≤ 0.01, *** indicates *p* ≤ 0.001, **** indicates *p* ≤ 0.0001, and ns indicates non-significant.

Altered mitochondrial respiration can be a consequence or can cause alterations to mitochondrial membrane potential and reactive oxygen species (ROS). As such, we measured mitochondrial membrane potential, mitochondrial superoxide, and hydrogen peroxide levels. Mitochondrial membrane potential was increased in female iNeurons and iAstrocytes from AD subjects (Figures 3A,B) but reduced in male cells derived from AD subjects. Mitochondrial superoxide was decreased in female iNeurons from AD subjects but increased in male iNeurons from AD subjects (Figure 3C). iAstrocytes showed an increase in mitochondrial superoxide in female cells derived from AD subjects, with no change in male derived cells (Figure 3D). iNeurons and iAstrocytes had an increase in hydrogen peroxide in female cells derived from AD subjects, but not in males (Figures 3E,F).

**Figure 4:**
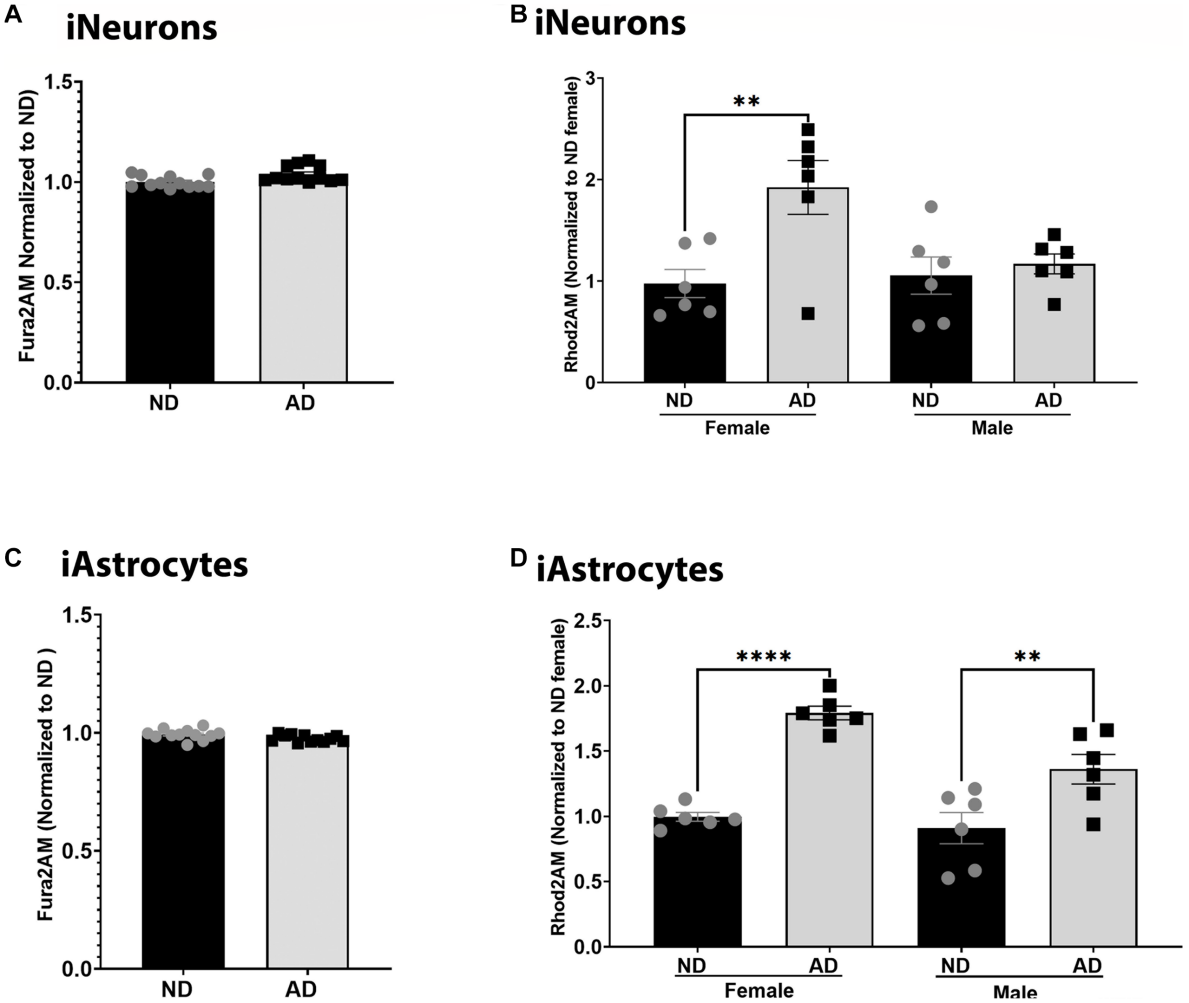
Calcium in iPSC derived models. **(A)** Fura2AM/Hoechst among ND and AD iPSC derived neurons, data are normalized to ND. **(B)** Rhod2AM/Hoechst among female and male groups of ND and AD iPSC derived neurons, data are normalized to female ND. **(C)** Fura2AM/Hoechst among ND and AD iPSC derived astrocytes, data are normalized to ND. **(D)** Rhod2AM/Hoechst among female and male groups of ND and AD iPSC derived astrocytes, data are normalized to female ND. ND, non-demented; AD, Alzheimer’s disease. * Indicates *p* ≤ 0.05, ** indicates *p* ≤ 0.01, *** indicates *p* ≤ 0.001, **** indicates *p* ≤ 0.0001, and ns indicates non-significant.

Calcium dynamics can profoundly change mitochondrial function and are critical to bioenergetic homeostasis. We measured total cellular calcium and mitochondrial calcium content. Total cellular calcium was not changed between ND and AD derived iNeurons and iAstrocytes (Figures 4A,C). However, iNeurons had an increase in mitochondrial calcium in female cells derived from AD subjects but not in males (Figure 4B). iAstrocytes showed increased mitochondrial calcium regardless of sex, in AD derived cells (Figure 4D).

**Figure 5:**
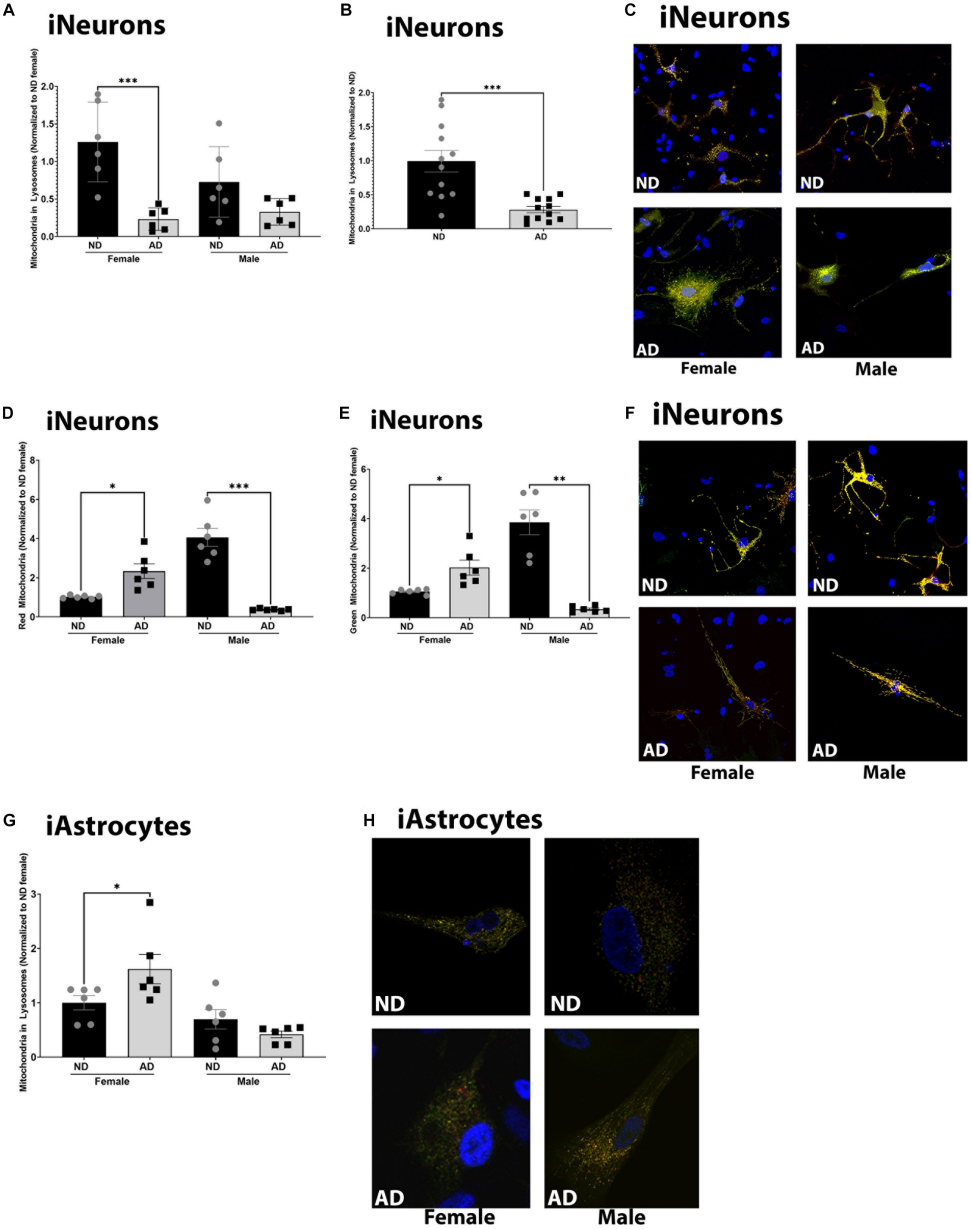
Mitochondrial turnover in iPSC derived models. **(A)** Mitochondria localized to lysosome per cell among female and male groups of ND and AD iPSC derived neurons, data are normalized to female ND. **(B)** Mitochondria localized to lysosome per cell among ND and AD iPSC derived neurons, data are normalized to ND. **(C)** Representative images from **(A,B)**. **(D)** Red, or mature mitochondria among female and male groups ND and AD iPSC derived neurons, data are normalized to female ND. **(E)** Green, or immature mitochondria among female and male groups ND and AD iPSC derived neurons, data are normalized to female ND. **(F)** Representative images from **(D,E)**. **(F)** Mitochondria localized to lysosome per cell among female and male groups of ND and AD iPSC derived astrocytes, data are normalized to ND. **(H)** Representative images from **(G)**. ND, non-demented; AD, Alzheimer’s disease. * Indicates *p* ≤ 0.05, ** indicates *p* ≤ 0.01, *** indicates *p* ≤ 0.001, **** indicates *p* ≤ 0.0001, and ns indicates non-significant.

Deficits in mitochondrial turnover through mitophagy and mitochondrial biogenesis have been noted in AD models. Here we measured basal mitophagy levels and mitochondrial biogenesis using adenoviral vectors (EFGP-mCherry COX8 and MitoTimer). The number of mitochondria localized to lysosomes was reduced in female iNeurons derived from AD subjects, but not in males (although a trend was present, Figures 5A–C). iAstrocytes derived from female AD subjects had increased mitochondria within lysosomes but no difference was observed between male AD and ND donors (Figures 5G,H). Overall, iNeurons from female AD subjects had an increase in the number of mature and immature mitochondria (Figures 5D–F). iNeurons derived from male AD subjects had a decrease in the number of mature and immature mitochondria (Figures 5D–F). iAstrocytes showed no differences in mitochondrial number, either mature or immature (data not shown).

**Figure 6:**
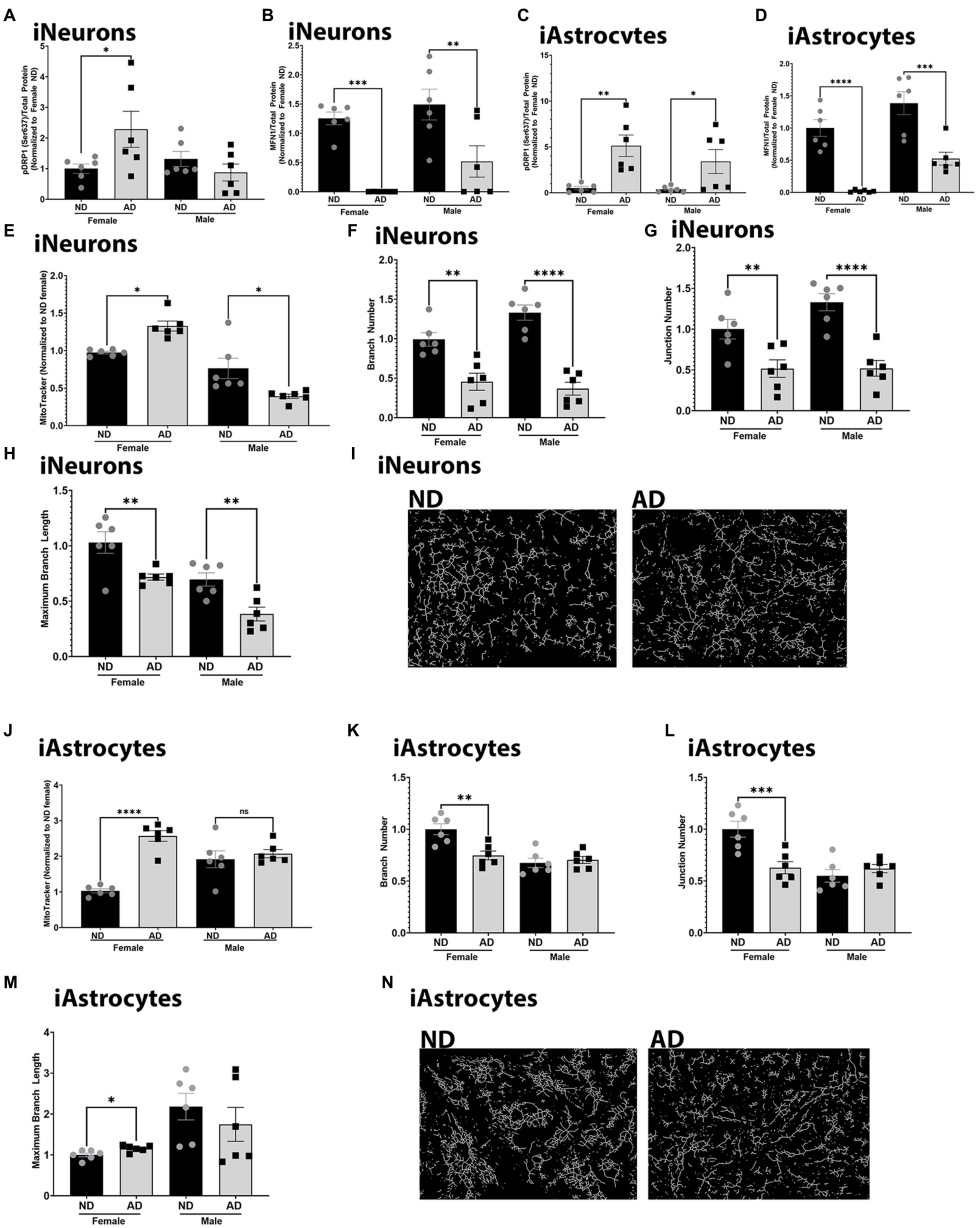
Mitochondrial dynamics in iPSC derived models. **(A)** Densitometry analysis of pDRP1 (Ser637) protein expression in iPSC derived neurons. **(B)** Densitometry analysis of MFN1 protein expression in iPSC derived neurons. **(C)** Densitometry analysis of pDRP1 (Ser637) protein expression in iPSC derived astrocytes. **(D)** Densitometry analysis of MFN1 protein expression in iPSC derived astrocytes. **(E)** MitoTracker intensity in iPSC derived neurons. **(F)** Mitochondrial branch number in iPSC derived neurons. **(G)** Mitochondrial junction number in iPSC derived neurons. **(H)** Maximum mitochondrial branch length in iPSC derived neurons. **(I)** Representative images of mitochondrial networks from iPSC derived neurons. **(J)** MitoTracker intensity in iPSC derived astrocytes. **(K)** Mitochondrial branch number in iPSC derived astrocytes. **(L)** Mitochondrial junction number in iPSC derived astrocytes. **(M)** Maximum mitochondrial branch length in iPSC derived astrocytes. **(N)** Representative images of mitochondrial networks from iPSC derived astrocytes. ND, non-demented; AD, Alzheimer’s disease. * Indicates *p* ≤ 0.05, ** indicates *p* ≤ 0.01, *** indicates *p* ≤ 0.001, **** indicates *p* ≤ 0.0001.

To further understand changes to mitochondria in iPSC derived AD models, we examined mitochondrial dynamics. Mitochondrial fission and fusion are critical for bioenergetic function and mitophagy. Both iPSC derived neurons and astrocytes from AD subjects had increased expression of phosphorylated DRP1 at Serine 637 (Figures 6A,C). There was no change in total expression of DRP1 (data not shown). iPSC derived neurons and astrocytes from AD subjects also had decreased MFN1 expression (Figures 6B,D). We did observe sex differences for phosphorylated DRP1 in the iPSC derived neurons, but not in astrocytes. MitoTracker intensity was increased in female derived iPSC neurons from AD subjects but decreased in those derived from male AD subjects (Figure 6E). iPSC derived neurons from AD subjects had reduced mitochondrial branch number, junction number, and maximum branch length (Figures 6F–I). We did not observe sex differences for these findings. MitoTracker intensity was increased in female iPSC derived astrocytes from AD subjects but no in those derived from male AD subjects. iPSC astrocytes derived from female AD subjects had reduced mitochondrial branch number, junction number, but increased maximum branch length (Figures 6K–N). These observations were not found in iPSC derived astrocytes from male AD subjects. Supplementary Figure S3 shows western blots for Figure 6.

**Figure 7:**
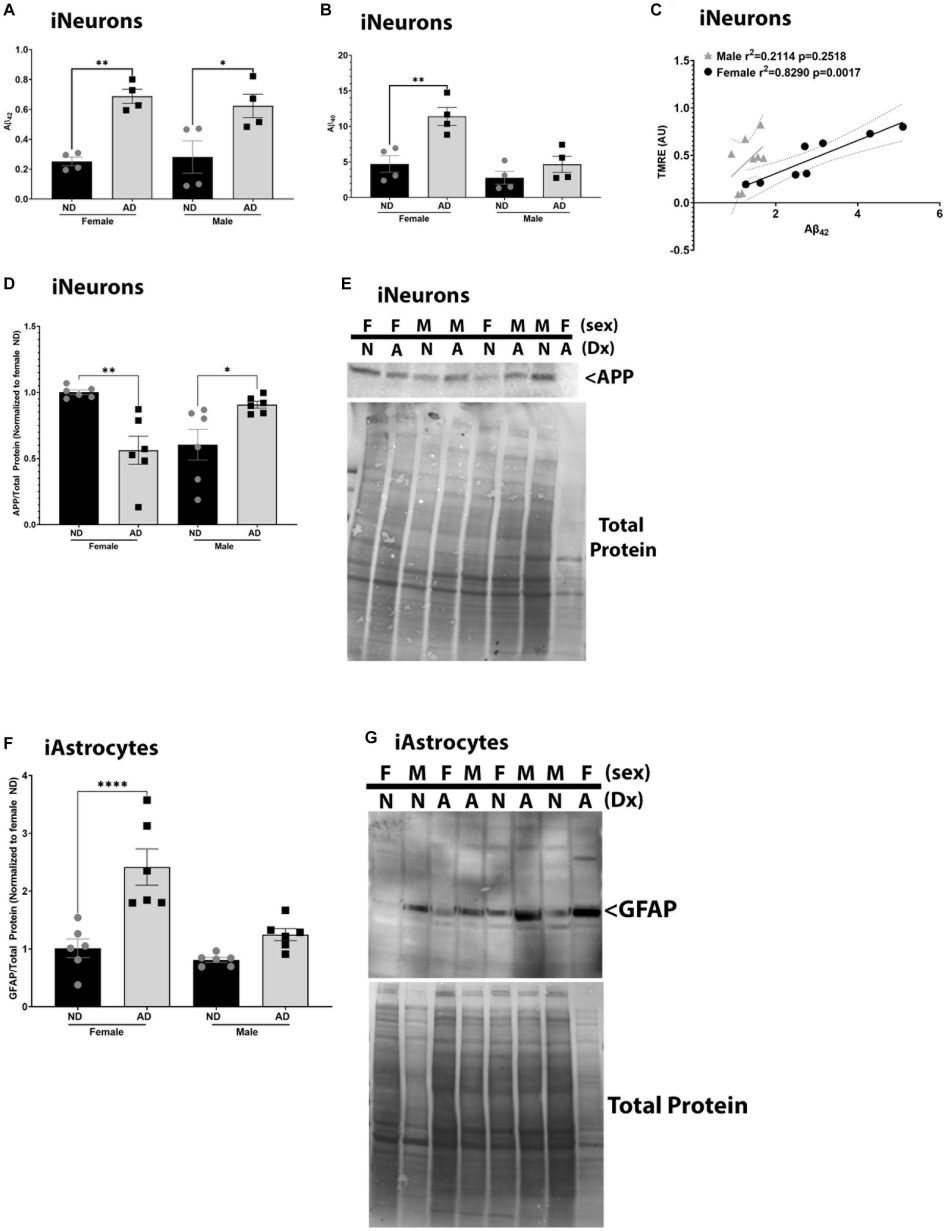
AD pathological hallmarks in iPSC derived models. **(A)** Secreted Aβ_42_ among female and male groups of ND and AD iPSC derived neurons, data are normalized to female ND. **(B)** Secreted Aβ_40_ among female and male groups of ND and AD iPSC derived neurons, data are normalized to female ND. **(C)** Correlation of mitochondrial membrane potential (TMRE) and secreted Aβ_42_ among female and male groups of ND and AD iPSC derived neurons. **(D)** APP protein expression among female and male groups of ND and AD iPSC derived neurons, data are normalized to female ND. **(E)** Representative western blot images for **(D)**. **(F)** GFAP protein expression among female and male groups of ND and AD iPSC derived astrocytes, data are normalized to female ND. **(G)** Representative western blots for **(F)**. ND, non-demented; AD, Alzheimer’s disease. * Indicates *p* ≤ 0.05, ** indicates *p* ≤ 0.01, *** indicates *p* ≤ 0.001, **** indicates *p* ≤ 0.0001, and ns indicates non-significant.

To determine if these models recapitulate phenotypes observed in AD, we measured Aβ, APP, and GFAP expression. We also examined relationships between mitochondrial membrane potential and Aβ, as we have previously described strong correlations between these outcomes (Wilkins et al., 2022). iNeurons derived from AD subjects had increased Aβ_42_ in media samples for both male and female cohorts (Figure 7A). Aβ_40_ was increased in media samples for female AD subjects only in iNeurons (Figure 7B). We had previously shown that mitochondrial membrane potential correlates with Aβ secretion, and this was recapitulated here in female derived iNeurons (Figure 7C) (Wilkins et al., 2022). Total full length amyloid precursor protein expression (APP) was decreased in female but increased in male AD derived iNeurons (Figures 7D,E) Finally, iAstrocytes derived from female AD subjects had an increase in glial fibrillary acid protein (GFAP) expression (Figures 7F,G).

## Discussion

iPSC derived models are gaining popularity in the AD research field. However, it is important to understand their phenotypes and nuances. Here we describe mitochondrial and AD pathological hallmarks in iPSC derived models. We considered cell type and sex specific differences, as these variables can and will impact study design. Most prior research has focused on neurons and have failed to consider sex differences. The importance of non-neuronal cell types in AD pathology have been noted and require further study. Furthermore, sex specific differences are imperative to understand given the increased risk of AD for females.

It is important to note that iPSCs from the KU ADRC clinical cohort are derived from fibroblasts upon autopsy which allows for a definitive diagnosis of AD with neuropathological exam. This is an important consideration in studies and allows for the possibility of linking clinical data with *in vitro* data. iPSCs purchased from WiCell were derived from fibroblasts but not upon autopsy, so a true definitive diagnosis of AD is not available (Israel et al., 2012). The gold standard for AD diagnosis is neuropathological exam at autopsy.

We observed mitochondrial phenotypes in iPSC derived neurons, astrocytes, and cerebral organoids in age and sex matched models derived from ND and AD subjects. Neuronal and cerebral organoid AD models showed reduced COX Vmax, while astrocytes did not. This finding is interesting and suggests a difference in physiology between single cell cultures and co-culture models. COX Vmax deficits have been noted across cell, animal, and human AD models (Kish et al., 1992; Curti et al., 1997; Cardoso et al., 2004; Crugnola et al., 2010). However, in iPSC derived models the finding of COX Vmax reduction in AD could be cell type and sex specific.

Mitochondrial and glycolytic flux were changed in both neuron and astrocyte derived models. Overall, we did not observe sex differences in these measures. The most significant finding is that both neuron and astrocyte derived models increase glycolytic flux and reduce mitochondrial flux. This suggests bioenergetic uncoupling and the possibility that these cell types could be competing for the same fuel sources (Fernandez-Fernandez et al., 2012; Qi et al., 2021). It is currently accepted that astrocytes provide bioenergetic support to neurons in the form of lactate, and that astrocytes typically produce more ATP from glycolysis while neurons leverage mitochondrial respiration for ATP production (Machler et al., 2016; Steinman et al., 2016; Muraleedharan et al., 2020; Yamagata, 2022). If both cell types are shifting to a more glycolytic state in AD, this could impair energy homeostasis and requires further investigation.

Both iPSC derived neurons and astrocytes had reduced ATP-linked respiration, consistent with an overall decrease in mitochondrial respiration. The iPSC derived neurons also had reduced leak rate, but as discussed below had increased indicators of ROS. These data suggest an overall uncoupling of mitochondrial respiration and potential reductions in redox homeostasis. Given that substrates are saturating in the Seahorse assays, this suggests that mitochondrial deficits are either intrinsic, i.e., enzyme deficits, or extrinsic, i.e., an inability of cells to transport substrates like glucose. Reductions in COX Vmax suggest intrinsic deficits in ETC enzyme structure and function, however future studies should examine glucose transport and uptake.

We also observed cell-type and sex specific changes to mitochondrial membrane potential and ROS levels. Mitochondrial membrane potential showed sex differences in both iPSC derived neurons and astrocytes, where female AD models were hyperpolarized, and male models were depolarized. This was consistent between cell types and could lead to altered ATP production and ROS. There were significant changes in mitochondrial superoxide and hydrogen peroxide levels in both neuron and astrocyte AD models, and these could reflect bioenergetic stress and failure of antioxidant enzyme pathways. We also observed an increase in mitochondrial calcium in female AD neuron and astrocyte models. Increased mitochondrial calcium is associated with bioenergetic stress, protein misfolding, and apoptosis (Jadiya et al., 2019; Calvo-Rodriguez et al., 2020; Ryan et al., 2020; Calvo-Rodriguez and Bacskai, 2021). Increased mitochondrial calcium has been noted across several AD models.

Mitophagy failure is a hallmark of AD and is recapitulated in iPSC models, however, there appear to be sex and cell-type specific differences. Both male and female AD subject iPSC derived neurons had reduced mitochondrial localization to lysosomes. The decreased mitochondrial lysosome content in the iPSC derived neurons was also supported by an increase in the total number of mitochondria, both mature and immature. In iPSC derived astrocyte models female derived AD cells had increased mitochondria localized to lysosomes while males showed no change. These results highlight potential cell type specific deficits in mitophagy pathways. Our data are consistent with findings of prior studies. Defects in lysosome degradation in fibroblast and iPSC models of familial AD have been described (Martin-Maestro et al., 2017). In postmortem human brain phosphorylated ubiquitin (pS65-Ub) levels are increased and localize with mitochondria (Hou et al., 2020). Other studies have indicated an increase in mitochondrial content in postmortem brain with possible increases in mitochondria within autophagic vesicles. These studies have been reviewed elsewhere recently (Mary et al., 2023). Overall, it’s not clear where the lesion is in mitophagy pathways in AD.

Mitochondrial dynamics are also altered in the iPSC derived models from AD subjects. Mitochondrial dynamics appear to be shifted away from fusion, with reductions in MFN1 expression and reduced mitochondrial branch lengths. Interactions between mitochondria also are reduced in both iPSC derived neurons and astrocytes from AD subjects. DRP1 phosphorylation is increased at Ser637, which is consistent with inhibition of fission (Knott et al., 2008). Given that the mitochondrial branch lengths are reduced the inhibition of fission through DRP1 could be a compensatory mechanism. Future studies should focus on understanding the context and consequences of these findings.

AD pathological hallmarks are observed in iPSC derived neurons. Secreted Aβ_40_ and Aβ_42_ were increased in AD neuron models with changes in total APP protein expression. iPSC derived neurons are not in culture for extended time periods and therefore do not develop Aβ plaques. Furthermore, GFAP expression in astrocyte AD models were higher in female cells. Sex differences in neuroinflammation phenotypes in AD have been noted previously in human subjects (Buckley, 2022; Coales et al., 2022). Prior studies have also shown that iPSC derived neurons from sporadic AD subjects have increase Aβ levels and phosphorylated tau (Ochalek et al., 2017).

iPSC studies leveraging female cells can be difficult due to unknown expression of maternal or paternal X chromosomes. This genetic variation could confound results, as different iPSC clones from the same donor could have an active maternal X chromosome or an active paternal X chromosome and this could yield varying phenotypes. However, our results indicate that sex as a biological variable is a critical component to consider in study design. While our study revealed interesting data, there are limitations. These include a limited number of iPSC donor cells and lack of power to examine effects of apolipoprotein E (*APOE*) genotype. However, our data highlight that future studies need to consider sex as a biological variable and be designed with enough power to detect sex differences. Consideration of sex differences is imperative for future therapeutic development and fundamental understanding of AD risk and cause.

Mitochondrial dysfunction is a hallmark of AD. Mitochondria are critical to proteostasis, and loss of proteostasis is an additional hallmark of AD. We have previously reported that mitochondrial membrane potential correlates with Aβ production and secretion, and these results were recapitulated here (Wilkins et al., 2022). The mitochondrial cascade hypothesis places mitochondria upstream of Aβ plaques and tau tangles in AD (Swerdlow et al., 1842). Supporting this hypothesis are studies which show direct relationships between mitochondrial function and Aβ and tau pathologies (Fukui et al., 2007; Pinto et al., 2013; Kukreja et al., 2014; Wilkins et al., 2015; Weidling et al., 2020; Wilkins et al., 2022). Overall, future studies should consider cell-type specific effects and work to understand the relationship between mitochondria and proteostasis in AD.

## Data availability statement

The raw data supporting the conclusions of this article will be made available by the authors, without undue reservation.

## Ethics statement

The studies involving human participants were reviewed and approved by the University of Kansas Medical Center Institutional Review Board. The patients/participants provided their written informed consent to participate in this study.

## Author contributions

HW conceptualized, obtained funding for the work, developed and performed assays, and wrote the manuscript. RS, JV, and MH edited the manuscript. KF, JS, BH, BT, CL, TS, VC, CAG, NS, JD, and CMG assisted with data collection. All authors contributed to the article and approved the submitted version.

## Funding

This study was supported by the Margaret “Peg” McLaughlin and Lydia A. Walker Opportunity Fund, Alzheimer’s Association Grant 23AARG-1023294, the Transgenic and Gene Targeting Institutional Facility at the University of Kansas Medical Center, the University of Kansas Cancer Center (NIH P30 CA168524) the University of Kansas Alzheimer’s Disease Center P30AG035982, R21TR003589, R00AG056600, and R01AG078186.

## Conflict of interest

The authors declare that the research was conducted in the absence of any commercial or financial relationships that could be construed as a potential conflict of interest.

## Publisher’s note

All claims expressed in this article are solely those of the authors and do not necessarily represent those of their affiliated organizations, or those of the publisher, the editors and the reviewers. Any product that may be evaluated in this article, or claim that may be made by its manufacturer, is not guaranteed or endorsed by the publisher.
